# Association of *MTNR1B* Gene Polymorphisms with Body Mass Index in Hashimoto’s Thyroiditis

**DOI:** 10.3390/ijms26083667

**Published:** 2025-04-12

**Authors:** Ivana Škrlec, Zrinka Biloglav, Davor Lešić, Jasminka Talapko, Igor Žabić, Darko Katalinić

**Affiliations:** 1 Faculty of Dental Medicine and Health, University J. J. Strossmayer Osijek, 31000 Osijek, Croatia; 2 Department of Medical Statistics, Epidemiology and Medical Informatics, School of Public Health Andrija Štampar, 10000 Zagreb, Croatia; 3 School of Medicine, University of Zagreb, 10000 Zagreb, Croatia; 4 Fizio Educa, 31000 Osijek, Croatia; 5 County Hospital Koprivnica, 48000 Koprivnica, Croatia; 6 Faculty of Medicine, Josip Juraj Strossmayer University of Osijek, 31000 Osijek, Croatia

**Keywords:** BMI, hypothyroidism, melatonin receptor, thyroid

## Abstract

Hashimoto’s thyroiditis (HT) is an autoimmune disorder of the thyroid gland characterized by chronic inflammation, which in most cases results in hypothyroidism. The melatonin receptor MTNR1B is sporadically expressed in the thyroid gland. It modulates immune responses, and alterations in the melatonin–MTNR1B receptor signaling pathway may play a role in developing autoimmune diseases. Obesity worsens the severity and progression of some autoimmune diseases and reduces treatment efficacy. This study aimed to investigate the association of *MTNR1B* gene polymorphisms (rs10830963, rs1387153, and rs4753426) with HT with regards to the body mass index (BMI). Patients with HT were categorized into normal weight BMI ≤ 25 kg/m^2^ and overweight/obese BMI > 25 kg/m^2^ groups. This study included 115 patients with a clinical-, ultrasound-, and laboratory-confirmed diagnosis of HT (64 normal-weight patients and 51 overweight/obese patients) with a mean age of 43 ± 12 years. The results showed that specific *MTNR1B* polymorphisms are associated with obesity in HT patients. BMI was found to be associated with the rs10830963 polymorphism, and the G allele and GG genotype of the rs10830963 polymorphism were more common in overweight/obese HT patients. Furthermore, the results suggest that genetic factors associated with BMI play a role in developing HT and open new possibilities for personalized treatment approaches.

## 1. Introduction

Hashimoto’s thyroiditis (HT), also known as chronic lymphocytic thyroiditis, is a common cause of hypothyroidism. This autoimmune disease affects 4.8–25.8% of women and 0.9–7.9% of men [1]. HT is characterized by lymphocytic infiltration of the parenchyma, causing a dense accumulation of lymphocytes, plasma cells, and macrophages as well as the presence of thyroid autoantibodies against thyroglobulin and TPO [2,3]. The infiltration of lymphocytes, especially T-cells, destroys the thyroid follicles and leads to gradual atrophy and fibrosis [4,5]. The elevated concentration of thyroid peroxidase (TPOAb) and/or thyroglobulin (TgAb) autoantibodies, together with the aforementioned hallmarks detected by a thyroid ultrasound [2,3,6], represent the characteristic features and valuable markers for HT diagnosis [7].

Circadian disruption also impairs the function of some endocrine glands, which can lead to altered melatonin secretion [8]. Melatonin regulates innate and adaptive immune responses and has been linked to autoimmune diseases [9,10,11,12]. Melatonin is the “hormone of darkness”, primarily synthesized and secreted by the pineal gland and the small intestine, liver, retina, lymphocytes, and melanocytes in the skin in both experimental animals and humans [13,14]. It has a pivotal role in sleep regulation and the light–dark cycle through various membrane and nuclear receptors, such as nuclear retinoid orphan receptors (ROR), Notch receptors, Toll-like receptors (TLR), and tumor necrosis factor receptors (TNF) [15,16]. Melatonin also modulates the circadian rhythm of immune responses against thyroid antigens and possesses anti-inflammatory effects to protect immune cells from oxidative stress [17,18]. Furthermore, melatonin suppresses the mitosis of thyroid follicular cells in vivo and thyroid hormone secretion [19,20,21]. Interestingly, in female rats, melatonin administration increases T4 levels [22], while in hypothyroid patients, a combination of melatonin and levothyroxine therapy reduces TSH and increases fT4 [23].

There are two primary types of melatonin receptors found in humans: MTNR1A (MT1) and MTNR1B (MT2) [24,25]. These G-protein-coupled membrane receptors are expressed in the thyroid gland [15] and various human tissues, including B and T lymphocytes [26,27]. Melatonin receptors MTNR1A and MTNR1B exhibit distinct expression patterns across tissues and organs, reflecting their specialized roles in physiological processes. *MTNR1B* is a glycemic-related gene sporadically expressed in the thyroid gland that encodes the MTNR1B receptor. Genetic variants in *MTNR1B* associated with fasting plasma glucose levels are described across different ethnic groups [28]. The MTNR1B receptor is also found in the immune system [26,29], including on CD4+ and CD8+ lymphocytes [30]. In monocytes, MTNR1B receptor expression depends on their maturation status [17], but there is no consensus regarding this process [25,31]. In animals, thyroid parafollicular C-cells can synthesize melatonin [32,33], suggesting that *MTNR1B* gene polymorphisms may increase the risk for autoimmune thyroid diseases [12]. Still, GWAS and other studies reported inconsistent associations between the *MTNR1B* locus and autoimmune thyroid diseases [9,10,11,12,34,35]. Melatonin’s interaction with MTNR1B receptors on immune cells may directly modulate immune responses [27,36,37,38]. A vicious cycle persists between disruption of the circadian rhythm and immune responses. Genetic alterations in the melatonin–MTNR1B receptor signaling pathway [9,10,11,12,39], linked to immune regulation, autoimmune diseases, and the thyroid [27,40], could affect autoimmune disease development and clinical phenotypes. Moreover, its high antioxidative properties could have therapeutic potential for thyroid disorders [41]. 

The etiology of autoimmune thyroid diseases includes complex interactions between numerous genetic and environmental risk factors. Among them, obesity contributes to the pathogenesis of HT and other autoimmune diseases [42,43] through chronic inflammation, adipokine secretion, and immune cell modulation [44,45]. A Mendelian randomization study addressed the importance of genetic attribution and the possible relationship between body mass index (BMI) [46] and autoimmune thyroiditis [47]. Obesity facilitates the progression of autoimmune diseases and reduces therapeutic effectiveness [48]. General and abdominal obesity increases the risk of HT, but this association is complex and bidirectional [49]. Furthermore, obesity may contribute to the onset and progression of HT and hypothyroidism, which leads to weight gain. 

The circadian rhythm disruption may be related to autoimmune thyroid diseases [50] and HT. Circadian rhythm influences T cell-mediated destruction of thyroid follicular cells and increases the production of pro-inflammatory cytokines [51], with TPOAb and TgAb exacerbating tissue damage [50]. Melatonin maintains a circadian rhythm via the MTNR1B receptor that controls immunological and metabolic processes [52]. Circadian rhythm disruptions increase TSH levels [53] by altering melatonin–MTNR1B signaling. In addition, obesity correlates with higher TSH concentrations and lower levels of thyroid hormones [54,55], and metabolic disturbances in obesity, such as dyslipidemia, overlap with the complications of hypothyroidism, creating a bidirectional relationship [53]. Genetic studies suggest that *MTNR1B* gene polymorphisms may be associated with the development of autoimmune diseases [9,10,11,12,51]. We analyzed three polymorphisms within the *MTNR1B* gene based on these potential associations. The rs10830963 polymorphism has been linked to BMI based on a GWAS study [56], obesity [57], and metabolic syndrome [58]. Although rs10830963 is an intron variant, it can affect splicing and gene expression. Tuomi et al. demonstrated that rs10830963 influences *MTNR1B* expression in the pancreas, particularly in human islet cells, increasing *MTNR1B* mRNA expression [59]. The rs1387153 polymorphism has been associated with Graves’ disease [12] and diabetes [60], as well as metabolic syndrome based on GWAS [61]. In addition, intragenic variants such as rs1387153 can occur in various enhancers and affect transcription factor binding and gene expression. Moreover, rs1387153 and rs10830963 are highly correlated (D′ = 0.827, r^2^ = 0.388), and the combination of these polymorphisms may affect *MTNR1B* expression. The polymorphism rs4753426 (−1193T > C) is located in the *MTNR1B* promoter and directly affects *MTNR1B* gene expression in the thyroid gland, and the CC genotype is associated with decreased *MTNR1B* gene expression in the thyroid [62].

However, a large knowledge gap exists between the association of *MTNR1B* polymorphisms and BMI in HT patients. Understanding the genetic basis of melatonin receptor function could provide insights into the molecular mechanisms underlying this prevalent autoimmune disorder. This study is the first one aimed at examining the association between *MTNR1B* polymorphisms and BMI in patients with Hashimoto’s thyroiditis. 

## 2. Results

In this study of HT patients (N = 115) with a mean age of 43 ± 12 years, 64 were of a normal weight, and 51 were overweight/obese. Table 1 shows the demographic and laboratory characteristics based on the BMI groups. The BMI > 25 kg/m^2^ group had lower fT4 concentrations that were marginally significant compared to the BMI ≤ 25 kg/m^2^ group (Z = −1.616; *p* = 0.050).

Table 2 shows the allele frequencies and distribution of genotypes of the *MTNR1B* polymorphisms. The G allele of the rs10830963 polymorphism was significantly more frequent in patients with a BMI > 25 kg/m^2^, and this association was statistically significant (χ^2^ = 5.173; *p* = 0.02). The association between the rs1387153 and rs4753426 polymorphisms and BMI did not reach statistical significance.

A logistic regression model with adjustment for confounders was created to estimate the effect of the selected polymorphism. There was a statistically significant association between the rs10830963 polymorphism and overweight/obesity. In the context of the recessive model, defined as GG versus CG + CC, the *p*-value was 0.031 (OR = 2.35; 95% CI 1.08–5.09). Furthermore, fT4 was also statistically significant when associated with overweight/obesity (*p* = 0.030), as shown in Table 3.

Table 4 shows the frequency of the codominant, dominant, and recessive genotype models in patients with a BMI ≤ 25 kg/m^2^ compared to those with a BMI > 25 kg/m^2^.

In Table 5, the linkage disequilibrium (LD) analysis of the rs10830963, rs1387153, and rs4753426 polymorphisms was represented by the relative coefficient of linkage disequilibrium (Lewontin’s D′ (|D′|)) and the correlation coefficient (r^2^) in Table 5. The rs10830963 and rs1387153 polymorphisms had the highest LD (D′ = 0.827), suggesting a small possibility of recombination between these polymorphisms. The lowest LD (D′ = 0.406) was between rs10830963 and rs4753426. According to the correlation coefficients, the population was closest to equilibrium for the rs10830963 and rs4753426 polymorphisms (r^2^ = 0.121). The most significant population deviation from equilibrium was between the polymorphisms rs10830963 and rs1387153 (r^2^ = 0.388).

The frequency of the predicted haplotypes based on the BMI categories are shown in Table 6.

Table 7 shows the association between the thyroid risk factors and tested SNPs based on BMI groups.

## 3. Discussion

This study confirms the association between the *MTNR1B* gene rs10830963 polymorphism and BMI > 25 kg/m^2^ in HT patients. The G allele and GG genotype of the rs10830963 polymorphism were more frequent in the overweight/obesity group of HT patients. The fT4 concentrations were associated with overweight/obesity in HT as measured in logistic regression. Disruptions in melatonin signaling, possibly due to polymorphisms in melatonin receptor genes, may dysregulate the immune system of HT patients [63,64]. 

The scarcity of previous studies limits the comparison with our findings. A large proportion of BMI variation is attributable to genetic factors, e.g., heritability in twin studies ranged from 40% to 70% [65,66]. Since obesity is a chronic, relapsing, multifactorial, neurobehavioral disease with complex genetic architecture, it is unlikely that any single genetic variant can explain the findings of the study. Obesity’s polygenic nature is influenced by the cumulative and small effects of over 500 obesity-related genes interacting with environmental factors [67]. Other characteristics among populations should be considered when discussing BMI [68,69]. Furthermore, the role of genetic factors in BMI appears to be more pronounced in women [68]. Sex differences have population relevance since HT is seven to ten times more prevalent in women [70]. 

As previously noted, melatonin regulates the wake–sleep cycle but also has a role in glucose metabolism, insulin sensitivity, and energy expenditure. We found an association between the rarer G allele of the rs10830963 polymorphism and the overweight/obesity group of HT patients. In general, most obese patients are metabolically “unhealthy” [71]. Metabolic syndrome is commonly associated with hypertension in HT patients with higher TSH concentrations and elevated levels of thyroid autoantibodies [72,73]. 

Hypothyroidism and slow metabolism, frequent clinical findings among HT patients, and the rs10830963 polymorphism can aggravate metabolic disorders and increase BMI. This polymorphism is associated with increased fasting glucose levels and a risk of type 2 diabetes, as well as decreased adiponectin levels [74]. A previous study suggested a link between the rs10830963 polymorphism and metabolic disorders, such as impaired blood glucose regulation and increased insulin resistance [75], which can lead to excessive fat storage and weight gain. 

An increased body weight, frequently associated with an increased risk of metabolic abnormalities, is strongly linked with thyroid dysfunction [76,77]. Thyroid cells release oxidases that catalyze the production of reactive oxygen species (ROS) [76,78], which damage the cells’ structure and the stability of the genome [76]. Melatonin’s protective effect against ROS-induced damage and obesity suggests that melatonin supplementation could be an effective intervention therapy for age-related diseases [79,80]. Sleep disturbances, such as insomnia, daytime sleepiness, or poor sleep quality, disrupt hormonal balance by increasing the appetite-stimulating hormone ghrelin, resulting in weight gain. Moreover, weight gain can also be attributable to stress associated with chronic thyroiditis and light disorders influences [50]. HT patients often have increased body weight compared to healthy individuals. An increase in fat mass during the hypothyroid phase usually normalizes with levothyroxine replacement therapy [81]. Although obesity is a known risk factor for hypothyroidism, the role of weight loss in risk reduction for hypothyroidism is still diminished [82]. 

Thyroid hormones can influence adipose tissue activity, and metabolic changes related to adipose tissue were observed in the deficiency or overproduction of thyroid hormones. While the effects of hypothyroidism and hyperthyroidism on body weight are well-documented, the evidence of the association between BMI and subtle variations in thyroid hormones in euthyroid patients is limited [83]. This study observed lower fT4 values that were borderline significant in overweight/obese HT patients. Sosa-López et al. reported a negative correlation between fT4 concentrations and the degree of obesity [84]. Similarly, Milionis et al. reported a negative correlation between fT4 concentrations and obesity [83]. Also, Milionis et al. reported no difference between serum fT3 concentrations in relation to BMI, nor did fT3 concentrations correlate with BMI [83]. 

In contrast, other studies reported that fT3 concentrations increase with increasing obesity [84,85]. Obese individuals, even though they were euthyroid, had higher fT3 concentrations than normal-weight individuals [85]. This study showed an association between the rs1387153 polymorphism and the fT3 and fT4 hormone concentrations in HT patients with a normal BMI. This polymorphism may modify melatonin receptor functionality and change the melatonin effect on the thyroid gland. Altered melatonin signaling could affect deiodinase enzymes responsible for converting T4 to the biologically active form of the hormone fT3, which may explain the association of fT3 concentrations with this polymorphism in individuals with HT. The fT3 level depends on the conversion of T4 to T3, a process that takes place in peripheral tissues (liver, kidneys). The rs1387153 polymorphism could affect the metabolic pathways involved in the conversion of these hormones, possibly through the modulation of insulin sensitivity, inflammation, or oxidative stress. The rs1387153 polymorphism could reduce the effect of melatonin, potentially increasing the inflammatory response in the thyroid gland and exacerbating thyroid tissue damage, which may directly affect fT3 concentrations [86]. The association of fT3 concentrations with the rs1387153 polymorphism in HT patients is likely the result of the interactions between the melatonin signaling pathway, thyroid regulation, and immune processes. These findings suggest a potential association between normal thyroid function and changes in BMI; however, the evidence is inconclusive [83]. The obesity category also affects changes in thyroid function, which may affect the normal range of thyroid hormone concentrations [84]. Changes in thyroid function are classified as primary, while changes in body weight are classified as secondary. The underlying cause may be singular or multifactorial, but the precise biological mechanisms are not fully understood. A possible hypothesis is a bilateral interaction between the thyroid gland and adipose tissue [48]. Variations in normal thyroid function are associated with changes in body weight. This study found no significant difference in TSH concentrations between patients with normal weight and those who were overweight/obese. In contrast, Milionis et al. reported significantly higher TSH concentrations in patients with a higher BMI but only in male hypothyroid patients, in whom a negative correlation between TSH and BMI was found [83]. The reported high TSH concentrations may be due to mild thyroid insufficiency, possibly exacerbated by obesity [87], as TSH correlates positively with BMI only in overweight women with elevated concentrations of thyroid autoantibodies [84,87,88].

### Limitations

In this study, we faced some limitations and strengths. A relatively homogeneously studied population in terms of age, ethnicity, and social environment represented the study’s major strength. The sample size represented the major limitation, but a convenient sampling frame was only possible during the usual clinical workup within the private medical institution. Such a sample size could have affected precision and reliability and may have increased variability. Consequently, the high odds ratios were represented with wide 95% confidence intervals. Additionally, some individual genotypes had low frequency, and genotype–phenotype associations should be interpreted cautiously. A larger sample would certainly provide a stronger genotype–phenotype association. Second, the analyzed polymorphisms may not be functionally associated with HT since functional polymorphisms would provide a non-confounded stronger association. This study focused on the *MTNR1B* gene polymorphisms and their associations with obesity, and we acknowledge the limitation of not including other gene variants that may also contribute to an increased BMI. 

Third, we did not measure serum melatonin concentrations, and the association between the *MTNR1B* polymorphisms and melatonin remains to be clarified. It should be noted that the maximum concentrations are individual-specific and age-dependent. After 25 or 40 years of age, melatonin production in the pineal gland drops to 60% of young adult levels. There is a steady decline in values as low as 20% of the young adult level in people over 90 years old, with average melatonin values always being higher in women [89,90]. 

Furthermore, it would be worthwhile investigating the relationship between *MTNR1B* gene polymorphisms, melatonin concentrations, and clinical severity of the disease. Fourth, since exposure and outcome are assessed simultaneously in cross-sectional studies, this study design is minimally informative for causal inference. Still, despite methodological limitations, it may provide insights into the causal effects of exposure to the disease [91]. Therefore, methodologically demanding longitudinal studies are needed to thoroughly elucidate the complex relationships between the *MTNR1B* gene polymorphisms, thyroid function, and body weight in HT patients. Fifth, the etiology of HT involves a complex interplay of genetic, environmental, and lifestyle factors, with genetic factors explaining only 5.5% of the genetic predisposition to date [92]. Additionally, epigenetic alterations contributing to the pathogenic mechanisms of the autoimmune disease were not analyzed in this study. This might contribute to a more accurate diagnosis of HT, appropriate choice in treatments, and a more precise prediction of treatment outcomes [93]. In clinical practice, the continuum of thyroid disorders is marked with two extreme conditions: hyperthyroidism and hypothyroidism. Awareness should be raised about the limitations of such bivariant perception and the complexity of interactions with various risk factors [94]. Since the melatonin–MTNR1B signaling pathway affects immune regulation, autoimmune diseases, and the thyroid, its potential role in HT should be considered. This study uniquely investigated the association between the *MTNR1B* polymorphisms in HT patients and the BMI categories. Despite the broadening of knowledge provided by the results, further research with more rigorous study designs is needed to ensure validation of the genetic variations underlying HT.

## 4. Materials and Methods

This cross-sectional study of HT patients (N = 115) was conducted from November 2023 to February 2024. This study was approved by the Ethics Committee of the private clinic Leptir Polyclinic Zagreb (No. 25-10-6-12/23, approved on 6 December 2023) and the Faculty of Dental Medicine and Health, Osijek (No. 2158/97-97-10-24-03, approved on 15 January 2024). This study was prepared following the principles of Good Clinical Practice, the Declaration of Helsinki, and all their amendments.

### 4.1. Participants

We included HT patients who had hypothyroidism diagnosed after the depletion of thyroid functional reserve; an echographic pattern of diffuse thyroid disease; and increased TSH, and/or decreased thyroid hormones—T3, T4 or fT4, and/or increased TPOAb and TgAb [95,96]. The inclusion criteria were above 18 years of age, and clinical-, ultrasound-, and laboratory-confirmed HT (positive TPOAb and positive TgAb, or positive TPOAb or positive TgAb). 

Exclusion criteria were: <18 years of age; euthyreosis; pregnancy; type 2 diabetes; malignancies; other autoimmune diseases; acute or chronic infectious conditions; oral supplementation of iodine, selenium, myoinositol, zinc, magnesium, iron, vitamin B complex, and vitamin D; treatment with hypolipemic drugs; specific dietary habits (vegetarians and vegans, consumers of turmeric or curcumin); obstructive sleep apnea (OSA); or other chronic and neurological diseases leading to sleep disorders. All analyses were determined at the same facility and with the same laboratory method to ensure the comparability of data. 

Medical history data included age, sex, height, and the following weight categories: normal weight (BMI ≤ 25 kg/m^2^) and overweight/obese (BMI > 25 kg/m^2^). 

### 4.2. Biochemical Analyses

Laboratory data included the values of total cholesterol, triglycerides, HDL (high-density lipoprotein), LDL (low-density lipoprotein), iron, vitamin D, TPOAb, TgAb, fT3, fT4, and TSH. The values for the Abbott chemistry sets (Abbott, Chicago, IL, USA) were used as reference values for the analytes listed: for cholesterol <5 mmol/L, for triglycerides <1.7 mmol/L, for HDL >1.2 mmol/L, for LDL <3 mmol/L, for iron from 8 to 30 µmol/L, for vitamin D 50 to 200 nmol/L, for TPOAb < 34 klU/L, for TgAb < 115 klU/L, for TSH 0.27 to 4.2 mU/L, for fT3 3.95 to 6.8 pmol/L, and for fT4 12 to 22 pmol/L.

The determination of TgAb, TPOAb, fT3, fT4, TSH, and vitamin D concentrations was performed by chemiluminescent microparticle immunoassay (CMIA) using Abbott reagent sets on an automated chemiluminescent analyzer Alinity i (Abbott Laboratories, Chicago, IL, USA). 

The iron concentration was determined by the photometric method with ferene, HDL by photometric method with cholesterol esterase, LDL by photometric method with cholesterol esterase and cholesterol oxidase, and triglycerides by the photometric method with lipase and glycerol-phosphate oxidase on an Alinity c automated device. Total cholesterol concentration was determined by the enzymatic method with cholesterol esterase and a set of chemicals manufactured by Abbott on an automated device called Alinity c (Abbott Laboratories, Chicago, IL, USA).

### 4.3. Genotyping

We analyzed the following *MTNR1B* polymorphisms: rs10830963 (chr11:92975544 (GRCh38.p14), C > G), rs1387153 (chr11:92940662 (GRCh38.p14), C > T), and rs4753426 (−1193 T > C, chr11:92968430 (GRCh38.p14), T > C).

The rs10830963 polymorphism is located in a single intron of *MTNR1B* and has no apparent effects on transcription factor binding or splicing [97]. The rs1387153 polymorphism is a non-coding polymorphism located 28 kb upstream of the 5’ region of *MTNR1B* on chromosome 11q21-q22. Global expression data from human lymphoblastoid cell lines did not indicate that rs1387153 directly affects *MTNR1B* expression [98]. The polymorphism rs4753426 (−1193T > C) is located in the promoter region of the *MTNR1B* gene and affects the transcriptional modulation of *MTNR1B* expression [62].

Genomic DNA extraction from whole blood was performed according to the manufacturer’s standard protocol using the QIAamp DNA Blood Mini Kit (Qiagen, Hilden, Germany). Polymorphisms of the *MTNR1B* gene were genotyped with the real-time polymerase chain reaction (qPCR) method on the QuantStudio 5 instrument (Thermo Fisher Scientific, Foster City, CA, USA) using the TaqMan method.

### 4.4. Statistical Analysis

SPSS (v 26.0, SPSS Inc., Chicago, IL, USA) was used for statistical data processing. Differences in the distribution and frequency of alleles were tested using the χ^2^-test. An analysis of individual polymorphisms according to different inheritance models (codominant, dominant, recessive) was performed using the logistic regression analysis approach within the SNPStats package [99] with genotype frequencies, proportions, odds ratios (OR), and 95% confidence intervals (CI). The allelic LD values of the three analyzed polymorphisms of the *MTNR1B* gene were analyzed using the program SHEsisPlus [100]. The values of the relative coefficient of linked disequilibrium (Lewontin’s D′ (|D′|)) and the correlation coefficient (r^2^) between each pair of polymorphisms were determined. Haplotypes were reconstructed using the PL-CSEM (Partition-Ligation Combination-Subdivision Expectation Maximization) algorithm, which is an integral part of the SHEsisPlus program [101]. The logistic regression model was utilized to ascertain the impact of the *MTNR1B* rs10830963, rs1387153, and rs4753426 genotypes on the probability of overweight/obesity, adjusted for the confounders: age, total cholesterol, triglyceride, HDL, LDL, vitamin D, iron, fT3, and fT4. The Mann–Whitney U test was utilized to ascertain the difference between the BMI groups concerning demographic and laboratory characteristics. The association between genotypes and risk factors was tested using the Kruskal–Wallis test. The significance level was set at a two-tailed significance level of *p* < 0.05, and all *p* values were adjusted according to the Bonferroni test for multiple testing.

The study’s power was calculated using the GAS Power Calculator [102]. Assuming a multiplicative model, a two-tailed significance level of 0.05, and a prevalence of the disease in the population at 0.02, the study’s sample size provided a power of 80% to detect significant ORs of 1.59, 1.58, and 1.57 for minor allele frequencies of 30%, 40%, and 50%, respectively, in the population with a normal BMI.

## 5. Conclusions

The G allele of the rs10830963 polymorphism of the *MTNR1B* gene and the GG genotype of the rs10830963 polymorphism were more frequent in overweight/obese HT patients than patients with a normal BMI. Still, we found no such difference for the other two polymorphisms, as the magnitude of a single gene on multifaceted clinical parameters, such as obesity, is generally low. Nevertheless, the *MTNR1B* gene polymorphisms could be considered as minor risk factors for obesity in HT patients. However, these adverse outcomes do not preclude the possibility that the analyzed polymorphisms might play a role in obesity in HT patients. Further studies are needed to elucidate the exact mechanisms underlying obesity in HT and to explore possible therapeutic interventions targeting the melatonin receptor signaling pathway.

## Figures and Tables

**Table 1:** Demographic and laboratory characteristics of HT patients.

Variable	BMI ≤ 25 (*n* = 64)	BMI > 25 (*n* = 51)	*p* *
	M ± SD	M ± SD	
Gender (female, N, %)	59 (92.2 %)	47 (92.2 %)	0.99
Age (years)	42.16 ± 12.61	43.08 ± 11.15	0.51
TPOAb (klU/L)	403.04 ± 372.79	468.44 ± 512.07	0.99
TgAb (klU/L)	221.18 ± 175.15	252.58 ± 215.89	0.72
TSH (mU/L)	12.43 ± 6.84	14.28 ± 8.29	0.11
fT3 (pmol/L)	5.31 ± 0.72	5.27 ± 0.88	0.84
fT4 (pmol/L)	15.90 ± 2.40	15.05 ± 2.55	**0.05**
Total cholesterol (mmol/L)	5.31 ± 1.02	5.34 ± 1.16	0.92
Triglycerides (mmol/L)	1.42 ± 1.07	1.34 ± 0.74	0.69
HDL (mmol/L)	1.84 ± 1.38	1.70 ± 1.23	0.35
LDL (mmol/L)	2.44 ± 1.01	2.42 ± 1.28	0.52
Iron (µmol/L)	15.76 ± 6.59	13.76 ± 7.29	0.11
Vitamin D (nmol/L)	58.83 ± 23.33	62.98 ± 21.31	0.28

BMI—body mass index, TPOAb—thyroid peroxidase antibodies, TgAb—thyroglobulin antibodies, TSH—thyroid-stimulating hormone, fT3—free triiodothyronine, fT4—free thyroxine, HDL—high-density lipoprotein, LDL—low-density lipoprotein, M—mean, SD—standard deviation. *p* *—*p* values from Mann–Whitney U tests, statistically significant values are in bold.

**Table 2:** Allele and genotype distribution and frequencies of the *MTNR1B* polymorphisms (N = 115).

SNP	Minor Allele	MAF BMI ≤ 25 (*n* = 64)	MAF BMI > 25 (*n* = 51)	χ^2^	*p* *	Genotype	BMI ≤ 25 (*n* = 64)	BMI > 25 (*n* = 51)	*p* *
rs10830963	G	0.398	0.549	5.173	**0.02**	CC	38 (59.4 %)	23 (45.1 %)	0.18
						CG	1 (1.6 %)	-	
						GG	25 (39.1 %)	28 (54.9 %)	
rs1387153	T	0.32	0.343	0.133	0.71	CC	32 (50 %)	24 (47.1 %)	0.94
						CT	23 (35.9 %)	19 (37.3 %)	
						TT	9 (14.1 %)	8 (15.7 %)	
rs4753426	T	0.484	0.421	0.902	0.34	CC	18 (28.1 %)	17 (33.3 %)	0.61
						CT	30 (46.9 %)	25 (49 %)	
						TT	16 (25 %)	9 (17.6 %)	

MAF—minor allele frequency. Data are presented as absolute and relative frequencies. *p* *—*p* values from Pearson’s chi-square tests, statistically significant values are in bold.

**Table 3:** Odds ratios for the HT patients’ overweight/obesity values adjusted for risk factors included in the logistic regression model.

Risk Factors	OR (95% CI)	*p*-Value
Age	1.01 (0.91–1.04)	0.717
fT3	1.44 (0.74–2.84)	0.287
fT4	0.79 (0.64–0.98)	**0.030**
Total cholesterol	0.93 (0.52–1.67)	0.808
Triglycerides	0.89 (0.56–4.41)	0.614
HDL	0.86 (0.61–1.25)	0.466
LDL	1.11 (0.64–1.93)	0.705
Iron	0.86 (0.89–1.02)	0.162
Vitamin D	1.01 (0.99–1.03)	0.310
rs10830963 (GG)	2.35 (1.08–5.09)	**0.031**

OR—odds ratio. Statistically significant *p* values are in bold.

**Table 4:** Models of *MTNR1B* gene genotypes based on BMI categories.

Genotypes Models		rs10830963	rs1387153	rs4753426
Codominant model	*p*	0.15	0.94	0.61
	OR	0.54	0.84	1.68
	95% CI	0.26–1.14	0.28–2.51	0.59–4.81
Dominant model	*p*	0.13	0.75	0.55
	OR	0.56	0.89	1.28
	95% CI	0.27–1.18	0.43–1.86	0.58–2.84
Recessive model	*p*	0.09	0.81	0.34
	OR	0.53	0.88	1.56
	95% CI	0.25–1.11	0.31–2.03	0.62–3.89

OR—odds ratio, CI—confidence interval.

**Table 5:** Linkage disequilibrium (LD) of the analyzed polymorphisms with the relative coefficients of linkage disequilibrium (D′) and correlation coefficients (r^2^).

		rs10830963	rs1387153	rs4753426
rs10830963	D′		0.827	0.406
	r^2^		0.388	0.121
rs1387153	D′			0.721
	r^2^			0.204

**Table 6:** Frequencies and distribution of probable *MTNR1B* haplotypes based on BMI groups.

rs10830963	rs1387153	rs4753426	Frequency BMI ≤ 25	Frequency BMI > 25	*p*
C	C	T	0.26	0.34	0.24
G	C	T	0.14	0.09	0.21
G	T	C	0.32	0.25	0.22
C	C	C	0.19	0.21	0.64

**Table 7:** Association of risk factors and SNPs of the *MTNR1B* gene based on BMI groups (N = 115).

Variable	rs10830963		rs1387153		rs4753426	
	BMI ≤ 25	BMI > 25	BMI ≤ 25	BMI > 25	BMI ≤ 25	BMI > 25
Gender	0.001	0.405	0.376	0.650	0.829	0.490
Age	0.176	0.691	0.693	0.822	0.453	0.587
TPOAb	0.256	0.339	0.482	0.230	0.705	0.611
TgAb	0.380	0.520	0.384	0.466	0.668	0.507
TSH	0.590	0.145	0.086	0.199	0.682	0.844
fT3	0.118	0.288	**0.044**	0.370	0.749	0.139
fT4	0.243	0.229	0.029	0.189	0.525	0.728
Total cholesterol	0.190	0.726	0.108	0.726	0.183	0.325
Triglycerides	0.316	0.441	**0.024**	0.524	0.173	0.779
HDL	0.704	0.510	0.562	0.194	0.098	0.626
LDL	0.250	0.501	0.464	0.550	0.901	0.489
Iron	0.739	0.338	0.763	0.457	0.919	0.087
Vitamin D	0.844	0.198	0.917	0.493	0.608	0.514

Statistically significant *p* values are in bold.

## Data Availability

The data presented in this study are available at the request of the corresponding author due to legal and ethical restrictions.
